# Genomic surveillance of *Anopheles* mosquitoes on the Bijagós Archipelago using custom targeted amplicon sequencing identifies mutations associated with insecticide resistance

**DOI:** 10.1186/s13071-023-06085-5

**Published:** 2024-01-04

**Authors:** Sophie Moss, Elizabeth Pretorius, Sainey Ceesay, Harry Hutchins, Eunice Teixeira da Silva, Mamadou Ousmane Ndiath, Robert T. Jones, Hristina Vasileva, Jody Phelan, Holly Acford-Palmer, Emma Collins, Amabelia Rodrigues, Sanjeev Krishna, Taane G. Clark, Anna Last, Susana Campino

**Affiliations:** 1 Faculty of Infectious and Tropical Diseases, London School of Hygiene & Tropical Medicine, London, UK; 2 Medical Research Council, The Gambia (MRCG), Fajara, Gambia; 3 Ministério de Saúde Pública, Bissau, Guinea-Bissau; 4 Projecto de Saúde Bandim, Bissau, Guinea-Bissau; 5 Clinical Academic Group, Institute for Infection and Immunity, St. George’s University Hospitals NHS Foundation Trust–St. George’s University of London, London, UK; 6 Centre de Recherches Médicales de Lambaréné (CERMEL), Lambaréné, Gabon; 7 Institut Für Tropenmedizin Universitätsklinikum Tübingen, Tübingen, Germany; 8 Faculty of Epidemiology and Population Health, London School of Hygiene & Tropical Medicine, London, UK

**Keywords:** Insecticide resistance, Vector control, Molecular monitoring, *Anopheles* mosquitoes

## Abstract

**Background:**

Insecticide resistance is reducing the efficacy of vector control interventions, consequently threatening efforts to control vector-borne diseases, including malaria. Investigating the prevalence of molecular markers of resistance is a useful tool for monitoring the spread of insecticide resistance in disease vectors. The Bijagós Archipelago (Bijagós) in Guinea-Bissau is a region of stable malaria transmission where insecticide-treated nets are the mainstay for malaria control. However, the prevalence of molecular markers of insecticide resistance in malaria vectors is not well understood.

**Methods:**

A total of 214 *Anopheles* mosquitoes were analysed from 13 islands across the Bijagós. These mosquitoes were collected using CDC light traps in November 2019, during the peak malaria transmission season. High-throughput multiplex amplicon sequencing was used to investigate the prevalence of 17 different molecular markers associated with insecticide resistance in four genes: *vgsc*,* rdl*,* ace1* and *gste2.*

**Results:**

Of the 17 screened mutations, four were identified in mosquitoes from the Bijagós: *vgsc* L995F (12.2%), N1570Y (6.2%) and A1746S (0.7%) and *rdl* A269G (1.1%). This study is the first to report the L995F knock-down resistance (kdr)-west allele in *Anopheles melas* on the Archipelago. An additional eight non-synonymous single-nucleotide polymorphisms were identified across the four genes which have not been described previously. The prevalences of the *vgsc* L995F and N1570Y mutations were higher on Bubaque Island than on the other islands in this study; Bubaque is the most populous island in the archipelago, with the greatest population mobility and connection to continental Guinea-Bissau.

**Conclusions:**

This study provides the first surveillance data for genetic markers present in malaria vectors from islands across the Bijagós Archipelago. Overall prevalence of insecticide resistance mutations was found to be low. However, the identification of the *vgsc* L995F and N1570Y mutations associated with pyrethroid resistance warrants further monitoring. This is particularly important as the mainstay of malaria control on the islands is the use of pyrethroid insecticide-treated nets.

**Graphical Abstract:**

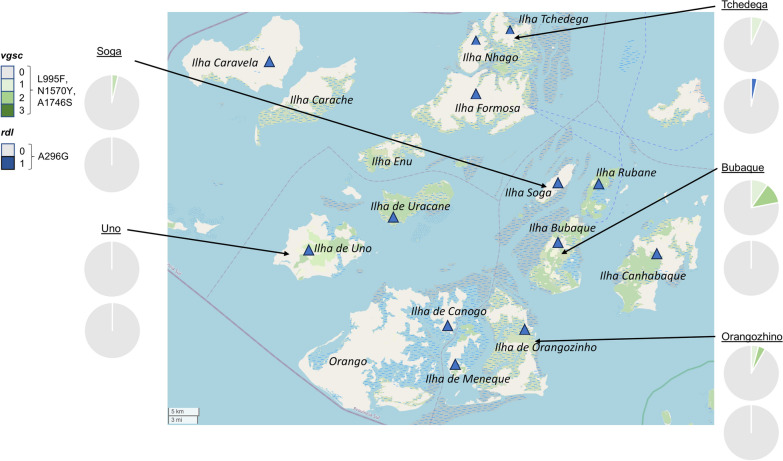

**Supplementary Information:**

The online version contains supplementary material available at 10.1186/s13071-023-06085-5.

## Background

Malaria still causes over 600,000 deaths every year, with around 95% of global cases occurring in Africa. Most of these cases are caused by the *Plasmodium falciparum* parasite, which is transmitted by *Anopheles* mosquitoes [1]. Vector control interventions are estimated to have reduced clinical malaria cases in Africa by over 600 million between 2000 and 2015 [2]. Of these interventions, insecticide-treated nets (ITNs) are estimated to have had the most impact, averting 68% of clinical malaria cases [2]. However, evolving insecticide resistance threatens the efficacy of vector control interventions. This presents  a threat to the control of all vector-borne diseases, including malaria [3]. Unfortunately, malaria vectors from all WHO malaria endemic regions have evolved resistance to all four classes of insecticides: pyrethroids, organochlorines, organophosphates and carbamates [4]. Consequently, the WHO recommends that countries routinely monitor insecticide resistance in malaria vectors to inform effective vector control policy [4, 5].

The WHO standard methodology for evaluating phenotypic resistance in vectors uses WHO tube tests, WHO bottle bioassays or CDC bottle bioassays [6]. These methods are time-consuming, and direct comparison between the results of CDC bottle bioassays and the WHO methods is not recommended. The WHO recognises that monitoring genetic mutations associated with insecticide resistance is valuable to resistance surveillance [4]. Furthermore, understanding the molecular mechanisms behind the evolution of resistance requires greater knowledge of the genomics underpinning resistance. Pyrethroid and dichlorodiphenyltrichloroethane (DDT) resistance has been associated with target-site mutations in the voltage-gated sodium channel gene (*vgsc*), also known as knock-down resistance, or kdr, mutations. This includes L995F (kdr-west) [7] and L995S (kdr-east) [8] (position 1014 in *Musca domestica*). In addition, metabolic resistance to pyrethroids and DDT has been associated with mutations in the glutathione s-transferase epsilon 2 gene (*gste2*), including L119F [9] and I114T [10]. Resistance to organophosphate and carbamate insecticides has been associated with the G280S mutation (formerly known as G119S) in the acetylcholinesterase 1 gene (*ace1*) [11], and organophosphate resistance has been linked with *ace1* gene duplications [12]. Mutations in the gamma-aminobutyric acid (GABA) receptor (*rdl*), including A296G have been associated with resistance to the organochlorine insecticide dieldrin [13].

The Bijagós Archipelago (Bijagós) is located 70 km off the coast of Guinea-Bissau, West Africa and includes 88 islands and islets, of which 18 are inhabited year-round. Malaria remains a key public health problem on the Bijagós, where malaria mortality increased between 2015 and 2021 [1]. The main component of malaria control in Guinea-Bissau is the use of ITNs, which have been distributed widely since 2011. Of the distributed ITNs in the Bijagós, 90% are PermaNet^®^ (Amabelia Rodrigues, personal communication), which are treated with deltamethrin, a pyrethroid insecticide. Coverage and use of ITNs on the Bijagós is reportedly high, with > 90% of 2018 survey participants reporting sleeping under a bed net [14]. No other vector control interventions are implemented on the islands, for example, indoor residual spraying, space spraying, or larviciding. The Bijagós Archipelago has a rainy season from June to October/November, and a dry season from December to May, with peak malaria transmission occurring in October and November. Two entomological surveys on the archipelago have previously been conducted in 2009 and 2017 [15, 16]. The major malaria vector has been identified as *Anopheles gambiae* sensu stricto (*A. gambiae *s.s.), which is the most prevalent Anopheline species during the onset of the rainy season in June/July. This contrasts to the end of the rainy season in November, and during the remainder of the dry season, where *Anopheles melas* has been found to be the predominant Anopheline species [16]. Guinea-Bissau is a particularly interesting location to study resistance evolution due to its high levels of *Anopheles gambiae* s.s.-*Anopheles coluzzii* hybridisation of > 20%, which may indicate the evolution of a novel hybrid form [17]. This unusually high level of recombination between species may provide the potential for the rapid spread of insecticide resistance alleles between *An. gambiae* s.s. and *An. coluzzii* populations.

The status of insecticide resistance in malaria vectors on the Bijagós Archipelago is largely unknown. Ant et al. [16] conducted an entomological survey of insecticide resistance on Bubaque island in 2017. This survey included CDC-bottle bioassays of *Anopheles gambiae* sensu lato (*A. gambiae* s.l.) mosquitoes, which suggested moderate resistance to α-cypermethrin and full susceptibility to permethrin. In the same study, Ant et al. [16] also investigated the presence of the *vgsc* L995F and L995S mutations, and found moderate frequencies of L995F in *An. gambiae *s.s. (36%), *An. coluzzii* (35%), and *An. gambiae/An. coluzzii* hybrids (42%). However, the presence of other mutations associated with insecticide resistance has not previously been investigated. Furthermore, the study by Ant et al. [16] was conducted with mosquitoes from Bubaque island only.

There have been recent advances in monitoring molecular markers of insecticide resistance with multiplex amplicon sequencing [18]. This method provides a high-throughput alternative to previous methods, such as Sanger sequencing or whole-genome sequencing, and uses primer barcoding to enable targeted sequencing of multiple genetic loci in many samples simultaneously, minimising required resources and lowering sequencing costs [18, 19]. In the present study, we aimed to conduct high-throughput molecular monitoring of insecticide resistance on the Bijagós, using mosquito samples collected from islands across the archipelago. Custom-targeted amplicon sequencing was used to investigate a panel of molecular markers associated with insecticide resistance across the *ace1, gste2, rdl* and *vgsc* genes. This approach was conducted using multiplex PCR assays and dual-indexing barcodes [18] to enable high-throughput sequencing of multiple loci across multiple samples.

## Methods

### Mosquito sampling and speciation

Mosquitoes were collected using indoor CDC miniature light traps (model 512; John W. Hock Company, Gainesville, FL, USA) using previously described methodology [20]. Mosquitoes were separated by genus, and *Anopheles* mosquitoes were morphologically identified using previously described keys [21]. All collected specimens were found to belong to *Anopheles gambiae* s.l. A subsample was then sent to the Medical Research Council Unit The Gambia (MRCG) at London School of Hygiene & Tropical Medicine for molecular analysis. Mosquito DNA was extracted and each sample was identified to species using PCR–restriction fragment length polymorphism (RFLP) [22].

### DNA extraction

DNA was extracted using the QIAamp^®^ 96 DNA QIAcube^®^ HT KIT (Qiagen, Hilden, Germany) with the QIA cube Extractor Robot, following the manufacturer instructions. DNA was eluted in 80 µl AE buffer and stored at − 20 °C.

### Species identification

Mosquitoes were identified to species using PCR–RFLP, based on the protocol of Fanello et al. [22] which uses primers to amplify the intergenic spacer (IGS) region to differentiate the members of the *An. gambiae* complex. The following primers were used: Universal F: GTGTGCCCCTTCCTCGATGT; *An. gambiae* R: CTGGTTTGGTCGGCACGTTT; *Anopheles arabiensis* R: AAGTGTCCTTCTCCATCCTA; *An. melas* R: TGACCAACCCACTCCCTTGA. Amplified PCR products were then digested using the HhaI enzyme to differentiate *An. gambiae* s.s. and *An. coluzzii* specimens. The band sizes of the PCR products were visualised by electrophoresis using the QIAxcel capillary electrophoresis system (Qiagen). The band sizes of the PCR products were analysed to distinguish species: *An. gambiae* s.s*.* (257 and 110 bp), *An. arabiensis* (292 bp)*, An. melas* (435 bp)*, An. coluzzii* (367 bp) and *An. coluzzii/An. gambiae* s.s*.* hybrid (257, 110 and 367 bp).

### Primer design

Primers were adapted from Campos et al. [18] to amplify seven regions of the *Anopheles* genome covering four different genes and 17 different single-nucleotide polymorphisms (SNPs) previously associated with insecticide resistance. Assays were designed to generate amplicons of approximately 500 bp. Primers were modified where possible to improve binding to the *An. melas* genome, using sequences downloaded from VectorBase (www.vectorbase.org). The primers used to amplify these regions are provided in Additional file 1: Table S2). The reverse primer for *gste2* was modified from Campos et al. [18] from TTCCAAATGCTTCCAAATTT to GGCTAGCACAAACTTGC. To enable multiplex amplicon sequencing, primers were further adapted in line with previously published methods to incorporate barcodes for multiplexing [18]. Briefly, 5ʹ barcode tags (8 bp long) were added to each forward and reverse primer (Additional file 1: Table S3). A total of 10 unique forward barcodes and 10 unique reverse barcodes were used. To enable multiplex sequencing, each mosquito sample was assigned a unique barcode combination prior to PCR amplification, resulting in the sample specific barcode being incorporated into the amplified PCR product.

### Multiplex PCR reactions

The Thermo Fisher Scientific (Waltham, MA, USA) multiplex primer analyzer was used to identify which primers were most suitable for multiplexing for amplicon generation. This resulted in multiplex 1 (*ace1* and *vgsc-3*), multiplex 2 (*rdl* and *vgsc-2*), multiplex 3 (*vgsc-4* and *gste2*) and a simplex reaction (*vgsc-1*). Each PCR reaction had a final reaction volume of 25 μl, containing 0.25 μl Q5 Hot-start DNA polymerase (New England Biolabs Ltd., Hitchin, UK), 5 μl of Q5 buffer (New England Biolabs Ltd.), 1 μl of DNA template (2–10 ng), 0.5 μl of 10 mM dNTPs, an average of 0.63 μl of each forward and reverse primer at 10 μM and 15.75 μl of nuclease-free H_2_O. PCR amplifications were conducted using the following reaction conditions: hot-start activation of Q5 Hot-start DNA polymerase for 30 s at 98 °C; followed by 35 cycles of denaturation at 98 °C for 10 s, annealing at 57 °C for 60 s and elongation 72 °C for 60 s; with a final elongation step of 72 °C for 2 min.

### Amplicon purification and next generation sequencing

Following the gene-specific multiplex PCR reactions, PCR products containing amplified amplicons were run in 1% agarose gels to detect the presence and the size of PCR products. The PCR products from different mosquito samples containing different barcodes were pooled. Pools were purified using KAPA pure beads (Roche Diagnostics, Indianapolis, IN, USA) to remove excess primers and PCR reagents at a ratio of 0.8:1 (μl of beads to μl of DNA). Purified pools were quantified using a Qubit before adjustment to a final concentration of 20 ng/µl in a final volume of 25 µl. Pooled samples were sent for sequencing at Genewiz GmbH (Leipzig, Germany) on the Illumina MiSeq platform paired-end (2 × 250 bp) configuration (Illumina Inc., San Diego, CA, USA), and a minimum of 50,000 reads were obtained per pool of amplicons. To obtain high sequencing coverage, a maximum of 200 amplicons were pooled per experiment, at a cost of < US$0.5 per amplicon.

### Bioinformatics analysis

Raw pooled FASTA sequences were de-multiplexed to separate sequences from individual mosquitoes through identification of sample-specific barcodes. Sequences were then processed to identify SNPs and insertions/deletions (INDELs) using a custom python script (https://github.com/LSHTMPathogenSeqLab/amplicon-seq). FASTA sequences were trimmed using Trimmomatic software [23] (version 0.39, using the parameters LEADING:3 TRAILING:3 SLIDINGWINDOW:4:20 MINLEN:36) to remove poor quality sequences. Paired-end reads were mapped to the *An. gambiae* reference genome (Anopheles_gambiae.AgamP4.dna.toplevel.fa) using BWA-MEM software (version 0.7.17-r1188, default parameters) to produce a BAM file for each mosquito. SNPs were called with Freebayes (version v1.3.6, haplotype-length-1) and GATK’s HaplotypeCaller (version 4.1.4.1, default parameters). Variants called by both variant callers were combined and filtered using BCFtools (version 1.16) for a minimum allele depth of 10, minimum read depth of 30 and phred score of > 30 per base. SnpEff software (version 5.1d) was used to annotate variants. Species-specific variants were removed to ensure these did not confound the analysis. Variants were genotyped based on the percentage of alternative allele reads within the total depth coverage: (i) homozygous reference (< 25% alternate allele calls); (ii) heterozygous (25–75% alternate allele calls); and (iii) homozygous alternate (> 75% alternate allele reads), as per Campos et al. [18]. SNPs were retained for analysis if they were present in more than one sample.

### Statistical tests

When comparing the allelic frequencies of alleles between island groups or species, statistical significance was investigated using a Chi-square (*χ*^2^)-test in R (df = 1) (version 4.1.0; R Foundation for Statistical Computing, Vienna, Austria).

## Results

### Mosquito species and geographic location

**Fig. 1:**
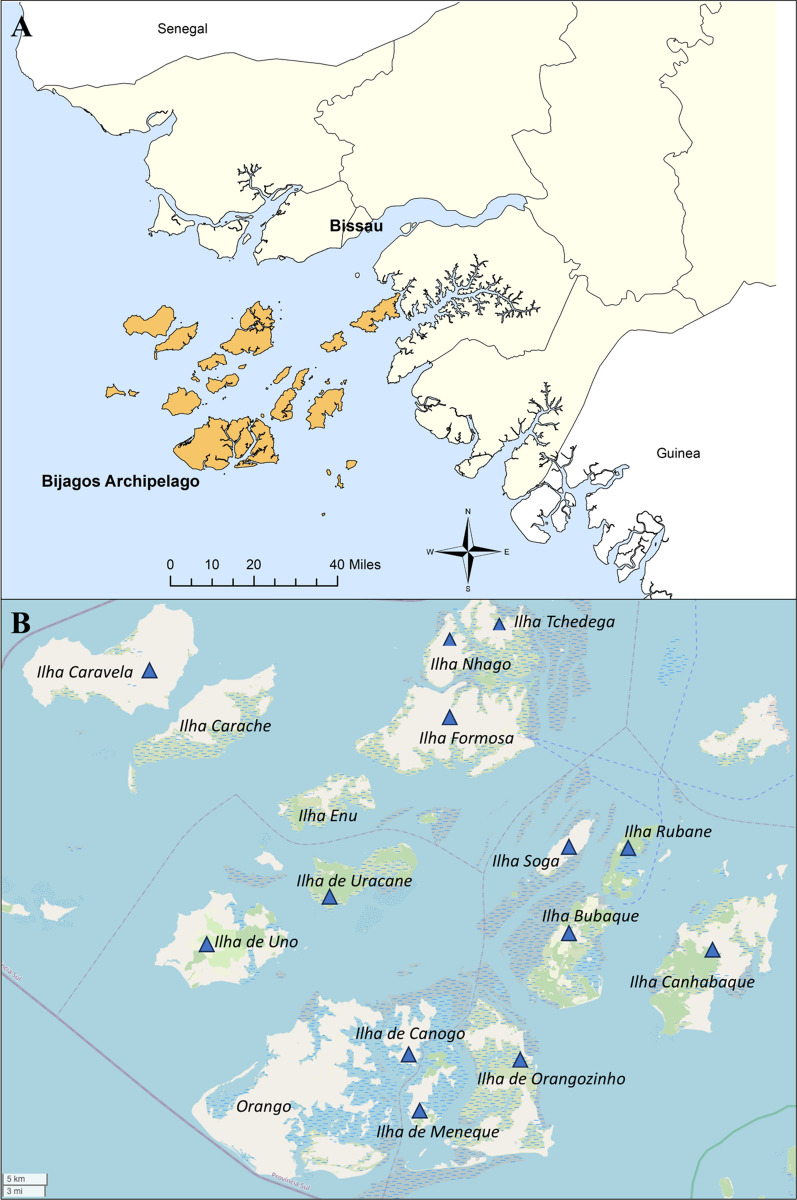
Mosquito sample collection sites. **a** Location of Bijagós Archipelago, created using ArcGIS ArcMap 10.8.1. **b** Mosquitoes were collected from the 13 islands labelled with blue triangles. Map from OpenStreetMap 2023-05-06

Mosquitoes were collected during a cross-sectional survey conducted on 16 islands of the Bijagós Archipelago during the peak malaria transmission season in November 2019. Of 409 mosquitoes collected, 82.2% were *An. melas,* 9.5% were *An. coluzzii-gambiae* hybrids, 6.82% were *An. gambiae* s.s. and 1.47% were *An. coluzzii* (Soubrier et al., unpublished data)*.* A subset of *n* = 214 *Anopheles* mosquitoes were used for analysis in this study. These mosquitoes were collected from 13 islands across the archipelago: Bubaque, Canhabaque (Roxa), Canogo, Caravela, Formosa, Tchedega, Meneque, Nhago (Ponta), Orangozinho, Rubane, Soga, Uno and Uracane (Fig. 1). The 214 mosquitoes included in this study are listed by the island on which they were collected and by species in Table 1. *Anopheles melas* was the most abundant species collected (113/214), with the majority of *An. melas *collected from Bubaque, Tchedega and Soga (70/113); these three islands accounted for 46% of all samples collected (99/214).

**Table 1:** Mosquitoes included in analysis and their island of origin in the Bijagós Archipelago

*Anopheles* species	Island													*Total*
	Bubaque	Canhabaque (Roxa)	Canogo	Caravela	Formosa	Tchedega	Meneque	Nhago (Ponta)	Orangozinho	Rubane	Soga	Uno	Uracane	
*An. melas*	24	2	1	10	0	24	2	7	0	5	22	12	4	*113*
*An. gambiae* sensu stricto	3	0	2	0	6	3	4	0	11	5	2	4	0	*40*
*An. coluzzii*	2	0	1	0	1	0	1	0	4	1	0	1	0	*11*
*An. coluzzii/An. gambiae* hybrids	12	3	5	1	4	3	1	0	9	3	4	4	1	*50*
Total	41	5	9	11	11	30	8	7	24	14	28	21	5	214

Species included *An. melas*, *An. gambiae* s.s.,* An. coluzzii*, and* An. coluzzii*–*An. gambiae hybrids*

### Amplicon sequencing coverage and detection of genetic variants

The presence of SNPs was investigated in each target gene. The SNPs targeted for amplification are listed in Additional file 1: Table S1. Amplicons were designed to cover these target SNPs and the surrounding gene region with a length averaging 500 bp, enabling the detection of SNPs in the neighbouring gene region (Additional file 1: Table S2). The average coverage obtained for each amplicon was very high (range: 210.43 to 632.91). This depth of coverage led to the identification of 155 high-quality SNPs across the seven amplicons. Of the 155 SNPs detected, 115 (74.2%) were in exons (12 non-synonymous SNPs and 103 synonymous SNPs), 28 (18.1%) were in introns, nine were upstream of exons and three were in splice regions (Table 2).

**Table 2:** Distribution of single-nucleotide polymorphisms detected in each gene

Gene^a^	Average coverage of amplicon (bp)	Synonymous (*n*)	Non-synonymous (*n*)	Intron (*n*)	Splice region (*n*)	Upstream gene variant (*n*)	Total (*n*)
*vgsc*-DIS6	600.81	1	2	16	0	0	19
*vgsc*-DIIS6	323.46	3	1	2	0	0	6
*vgsc*-DIIIS6	597.83	5	1	5	1	0	12
*vgsc-*DIVS5	210.43	5	1	5	1	0	12
*rdl*	253.54	2	1	0	0	0	3
*gste2*	336.72	22	4	0	1	9	36
*ace1*	632.91	65	2	0	0	0	67
Total		103	12	28	3	9	155

^a^Four amplicons are in the voltage-gated sodium channel gene (*vgsc* I-IV, D = domain, S = subunit). One amplicon is in each of the gamma-aminobutyric acid (GABA) receptor gene (*rdl*), glutathione s-transferase epsilon 2 gene (*gste2*) and acetylcholinesterase 1 gene (*ace1*)

The position and frequency of detected non-synonymous variants is summarised Table 3.

**Table 3:** Position and frequency of detected non-synonymous single-nucleotide polymorphisms in mosquitoes sampled from the Bijagós Archipelago

Chromosome	Amplicon	Position	*N*^a^	Mutation	Genotype frequencies (%)			Alternate allele frequency (%)
					Homozygous (reference)	Heterozygous	Homozygous alternate	
2L	*vgsc-*I	2391191	159	F389L	98.7	1.3	0.0	0.6
		2391309	160	E429K	58.1	41.9	0.0	20.9
	*vgsc-*II	2422652	135	L995F^b^*	82.2	11.1	6.7	12.2
	*vgsc-*III	2429745	170	N1570Y^b^	88.8	10.0	1.2	6.2
	*vgsc-*IV	2430424	139	A1746S^b^	98.6	1.4	0.0	0.7
2L	*rdl*	25429236	89	A296G^b^	97.8	2.2	0.0	1.1
2R	*ace1*	3491844	175	P203Q	98.9	1.1	0.0	0.6
		3492205	175	E323D	98.3	1.1	0.6	1.1
3R	*gste2*	28597858	182	I187F	98.9	1.1	0.0	0.5
		28597896	182	P174L	48.4	2.7	48.9	50.3
		28597905	182	G171D	89.6	9.3	1.1	5.8
		28597956	189	T154S	69.3	21.7	9.0	19.8

^a^N is the total number of alleles called for each position

^b^This mutation was one of the 17 targeted insecticide resistance single-nucleotide polymorphisms

Two non-synonymous SNPs were identified in the *vgsc-*DIS6 amplicon: Phe389Leu (F389L) and Glu429Lys (E429K). F389L was found at an allelic frequency of 0.6%, and E429K was found at an allelic frequency of 20.9%. These SNPs have not previously been associated with insecticide resistance. One non-synonymous SNP was identified in the *vgsc-*DIIS6 amplicon, namely the Leu995Phe (L995F) mutation, which is also known as the kdr-west allele. This mutation was found at an allelic frequency of 12.2% in the total population of mosquitoes sampled, and has previously been associated with resistance to pyrethroids and DDT [7]. One non-synonymous mutation, Asn1570Tyr (N1570Y), was identified in the *vgsc-*DIIIS6 amplicon, at an allelic frequency of 6.2%. The N1570Y mutation has been associated with increased levels of pyrethroid resistance when associated with L995F [24]. One non-synonymous SNP was identified in the *vgsc-*DIVS5 amplicon, Ala1746Ser (A1746S), with an allelic frequency of 0.7%. A1746S has previously been detected in *Anopheles* *gambiae* s.l. mosquitoes in Guinea and Ivory Coast [18, 25], and has been identified as a variant with a potential functional role in pyrethroid resistance [25]. One non-synonymous SNP, Ala296Gly (A296G), was identified in the *rdl* amplicon at an allelic frequency of 1.1%. A296G has previously been associated with resistance to dieldrin [13]. Two non-synonymous mutations were identified in the *ace1* amplicon, namely Pro203Gln (P203Q) at 0.6% and Glu323Asp (E323D) at 1.1%. These two SNPs have not previously been associated with insecticide resistance. Four non-synonymous SNPs were identified in the *gste2* amplicon: Ile187Phe (I187F), Pro174Leu (P174L), Gly171Asp (G171D) and Thr154Ser (T154S); these mutations were found at allelic frequencies of 0.5%, 50.3%, 5.8%, and 19.8%, respectively, and have not previously been associated with insecticide resistance.

### Insecticide resistance SNP distribution per island

Overall, four of the targeted SNPs (Additional file 1: Table S1) were identified in the samples collected on the Bijagós, including three mutations in the *vgsc* gene (L995F, N1570Y and A1746S) and one mutation in the *rdl* gene (A296G). The frequency of these mutations per island was investigated (Table 4). The L995F mutation was found at an overall frequency across the archipelago of 12.2% and was present in mosquitoes from 10 of the 13 islands. The N1570Y mutation was present at a frequency of 6.2% (present in 11/13 islands) and the A1746S mutation was present at a frequency of 0.7% (present in 2/13 islands). The *rdl* A269G mutation had a frequency of 1.1% (present in 2/13 islands).

**Table 4:** Allelic frequency of insecticide resistance single-nucleotide polymorphisms per island

Island	*N*^a^	Allelic frequency of insecticide resistance mutation (%)^b^			
		*vgsc* L995F	*vgsc* N1570Y	*vgsc* A1746S	*rdl* A296G
Bubaque	41	26.0	11.0	1.9	–
Canhabaque	5	33.0	12.5	–	–
Canogo	9	14.3	6.3	–	–
Caravela	11	8.3	5.6	–	–
Formosa	11	31.3	16.7	–	5.6
Tchedega	30	3.3	2.1	–	10.0
Meneque	8	20.0	14.3	–	–
Nhago	7	–	–	–	–
Orangozinho	24	7.7	2.9	–	–
Rubane	14	11.1	4.2	5.6	–
Soga	28	–	2.2	–	–
Uno	21	–	–	–	–
Uracane	5	12.5	12.5	–	–

^a^Total number of mosquitos sampled from each island

^b^The proportion of alleles that harbour any of the four identified target mutations

**Fig. 2:**
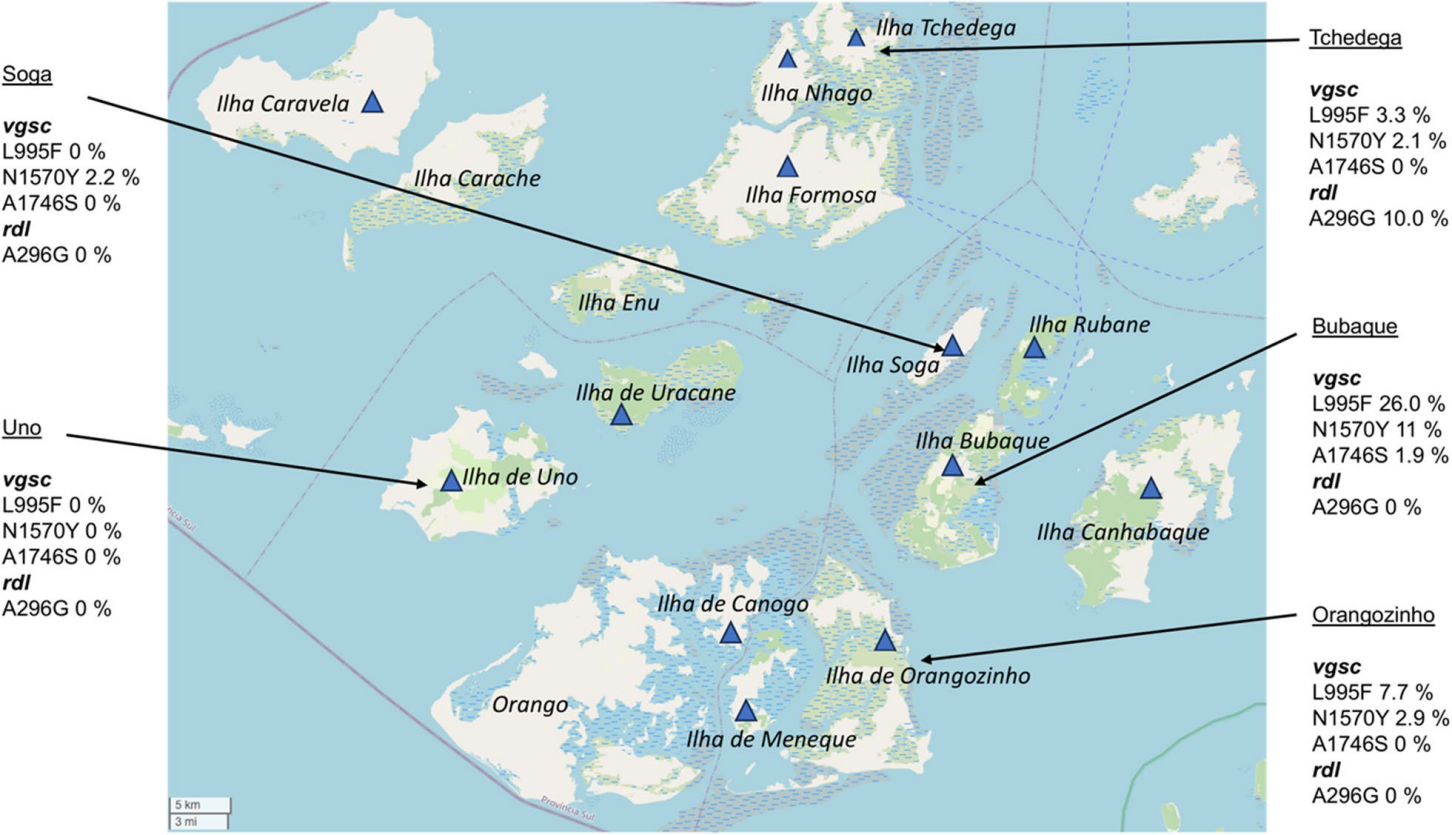
Allelic frequencies of the insecticide resistance mutations identified, mapped to island geographic location. Only islands with a sample size > 20 sampled mosquitoes were included in this analysis. *rdl*, Gamma-aminobutyric acid receptor gene; *vgsc*, voltage-gated sodium channel gene

Five of the islands were represented by > 20 mosquitoes: Bubaque (*n* = 41), Tchedega (*n* = 30), Orangozinho (*n* = 24), Soga (*n* = 28) and Uno (*n* = 21). The allelic frequencies of the four insecticide resistance mutations identified were mapped to island location (Fig. 2), and differences assessed. Bubaque had a significantly higher allelic frequency of *vgsc* L995F than Tchedega (*P* = 0.023), Soga (*P* = 0.001) and Uno (*P* = 0.008), and a slightly higher L994F frequency than Orangozinho, but this increase was not significant (*P* = 0.110). Bubaque had a significantly higher allelic frequency of *vgsc* N1570Y than Tchedega (*P* = 0.016) and Uno (*P* = 0.031). Bubaque had a slightly higher frequency of *vgsc* N1570Y than Orangozhino and Soga, but these differences were not significant. There was no statistical significance between the allelic frequency of the *vgsc* A1746S mutation between islands. The *rdl* A296G mutation was found on Formosa and Tchedega islands only.

**Fig. 3:**
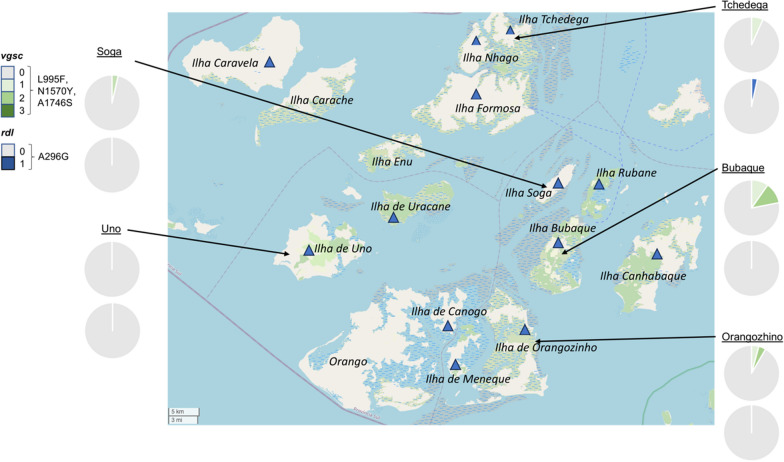
Distribution of cumulative insecticide resistance mutations across five islands on the Bijagós Archipelago. Only regions with > 20 sampled mosquitoes are shown (Bubaque, Orangozinho, Tchedega, Soga and Uno). The top pie chart for each island represents the percentage of samples with 0, 1, 2 or 3 of the targeted *vgsc* mutations, with the *vgsc* mutations shown in green. The lower pie chart for each island represents the percentage of samples with 0 or 1 *rdl* mutation, with the *rdl* mutations shown in blue. *rdl*, Gamma-aminobutyric acid receptor gene; *vgsc*, voltage-gated sodium channel gene

The number of different samples which had one or multiple insecticide resistance SNPs were investigated (Fig. 3). A small number of mosquitoes were found to have more than one resistance-associated mutation. This included five mosquitoes from Bubaque island, which had two *vgsc* mutations (L995F and N1570Y, or L995F and A1746S), and one mosquito from Orangozinho which had two *vgsc* mutations (L995F and N1570Y). All other mosquitoes had only one of the four identified insecticide resistance mutations.

### Distribution of insecticide resistance SNPs per mosquito species

The distribution of insecticide resistance SNPs between different *Anopheles* species samples was investigated (Table 5). The total sample set contained 214 mosquitoes: 113 *An. melas*, 40 *An. gambiae* s.s*.*, 11 *An. coluzzii* and 50 *An. coluzzii-An. gambiae* hybrid mosquitoes.

**Table 5:** Distribution of the four identified insecticide resistance single-nucleotide polymorphisms by *Anopheles* species

*Anopheles* species	*N*^a^	Allelic frequency of insecticide resistance mutation (%)			
		*vgsc* L995F	*vgsc* N1570Y	*vgsc* A1746S	*rdl* A296G
*An. melas*	113	2.14	1.12	0.76	0
*An. gambiae* sensu stricto	40	14.29	6.45	1.67	1.67
*An. coluzzii*	11	25.0	14.29	–	–
*An. coluzzii-An. gambiae* hybrids	50	21.1	13.1	–	1.25

^a^*N* is the total number of mosquitoes collected from each species

The *vgsc* L995F mutation was found in 24 mosquitoes, and across species (3 *An. melas*, 7 *An. gambiae* s.s*.*, 2 *An. coluzzii*, 12 *An. coluzzii-An. gambiae* hybrids). The *vgsc* N1570Y mutation was found in 18 mosquitoes (2 *An. melas*, 4 *An. gambiae* s.s., 2 *An. coluzzii* and 10 *An. coluzzii-gambiae* hybrids). The *vgsc* mutation A1746S was found in two mosquitoes (one *An. melas* and one *An. gambiae *s.s). Similarly, the *rdl* A296G mutation was found in only two mosquitoes (1 *An. gambiae *s.s. and 1 *An. coluzzii-gambiae* hybrid). Interestingly, *An. melas* had significantly lower allelic frequencies of *vgsc* L995F and N1570Y than the other mosquito species (*P* < 0.05). There was no statistically significant difference between the allelic frequencies of different species for *vgsc* A1746S or *rdl* A296G.

## Discussion

Vector control interventions, in particular ITNs, are the most effective method of reducing deaths due to malaria to date [2]. The emergence and spread of insecticide resistance is a growing threat to the utility of these interventions. Monitoring the prevalence of genomic polymorphisms associated with insecticide resistance is an important tool for monitoring the distribution and spread of insecticide resistance, and to complement phenotypic bioassays of resistance [18, 26, 27]. Monitoring polymorphisms can provide early warning signals to disease control programmes that resistance markers are changing. In turn, this information may inform evidence-based decisions in vector control policy. This is particularly useful in situations where resistance is present at low frequency and may be difficult to detect using phenotypic bioassays.

Multiplex amplicon sequencing is a high-throughput, low-cost and resource-efficient method of investigating the frequency of SNPs in sample populations. This technique has been used widely to investigate the frequency of insecticide resistance mutations in mosquito populations [18, 26–28]. We employed this technique to conduct the first investigation of multiple genomic markers of insecticide resistance across islands of the Bijagós Archipelago. We investigated the presence of genetic markers, including 17 SNPs associated with insecticide resistance in the *vgsc, gste2, rdl* and *ace1* genes. Four of the 17 targeted SNPs were identified in the Bijagós mosquito population. These were *vgsc* L995F and N1570Y, which are associated with DDT and pyrethroid resistance [7, 8], *vgsc* A1746S, which has a potentially functional role in pyrethroid resistance [25], and the *rdl* A296G mutation associated with dieldrin resistance [13].

The *vgsc* L995F (kdr-west) mutation was found at an allelic frequency of 12.2% across the archipelago and a frequency of 26.0% on the island of Bubaque. This was lower than previously identified by Ant et al. in a 2017 survey on Bubaque island, which found frequencies of 35% for *An. coluzzii,* 36% for *An. gambiae* s.s. 42% for *An. gambiae* s.s.-*An. coluzzii* hybrids [16]. However, our study methods had the capacity to distinguish between homozygous and heterozygous calls, which is likely to explain this discrepancy in allele frequency. A separate survey in Guinea-Bissau in 2018 by Silva et al. [29] also found higher frequencies of the L995F mutation (31.29%) in the nation’s capital, Bissau. We found the *vgsc* N1570Y mutation at an allelic frequency of 6.2% across the archipelago. This mutation has been referred to as the ‘super-kdr’ mutation and is predicted to intensify L995F-mediated pyrethroid resistance and/or compensate for the deleterious fitness effects of L995F [24]. The N1570Y mutation has been found previously in Bissau at a similar frequency of 6.62% [29]. The *vgsc* A1746S mutation, which has previously been identified in Guinea and Ivory Coast [25], was found in the present study at 0.7% allelic frequency. Two additional non-synonymous SNPs were found in the *vgsc* gene, Phe389Leu (F389L) (0.6%) and E429K (20.9%); these mutations have not previously been reported.

The A296G mutation in the *rdl* gene associated with resistance to dieldrin [13] was found at an allelic frequency of 1.1%. No additional non-synonymous SNPs were identified in this gene. There were two non-synonymous SNPs identified in the *ace1* gene: P203Q (0.6%) and E323D (1.1%); these SNPs have not been reported previously. The G280S mutation (formerly G119S) is associated with resistance to carbamates and organophosphates [30], and was not found in the Bijagós mosquito population. This mutation was also not found by Silva et al. [29] in Bissau, but it was found at other sites on the mainland, including Gabu, the easternmost region of Guinea-Bissau. Four non-synonymous mutations were found in the *gste2* amplicon which have not previously been reported: I187F (0.5%), P174L (50.3%), G171D (5.8%) and T154S (19.8%). The I114T and L119V mutations associated with pyrethroid [9] and DDT resistance [9, 10], and the F120L mutation associated with DDT resistance [31], were not identified in the Bijagós mosquito population. A notably larger number of SNPs were identified in the *ace1* and *gste2* amplicons than in the *vgsc* or *rdl* amplicons. This may be due to copy number variations, which are well-reported in the *An. gambiae* complex to result in multiple copies of *ace1* and *gste2* alleles [32, 33]. The presence of multiple copies may allow the accumulation of mutations in one copy of an allele without leading to deleterious fitness effects due to the presence of additional allele copies, enabling the accumulation of greater numbers of mutations over time.

We compared the frequency of insecticide resistance SNPs on five islands: Bubaque, Tchedega, Orangozinho, Soga and Uno. The frequency of L995F was significantly higher on Bubaque Island than on Tchedega, Soga or Uno, and slightly higher than on Orangozinho. Bubaque is the most populated island, home to around 28% of the total population of the archipelago (Ministério da Saúde Pública, Guinea-Bissau). It is also the site of the main ferry port to Bissau. Therefore, it is likely that there is a higher density of pyrethroid ITNs on Bubaque, resulting in greater selection pressure for the L995F mutation. Furthermore, the ferry link to mainland Guinea-Bissau provides a regular opportunity for ‘hitch-hiking’ mosquitoes carrying resistance alleles to be transported to Bubaque. This hitch-hiking may result in a larger number of resistance alleles in the Bubaque mosquito population for positive selection to act upon. The sample size of mosquitoes from each island was limited and should be increased in future work to enable better comparisons of allele frequency between islands.

We compared the frequency of insecticide resistance SNPs between species and found that *An. melas* had significantly lower allelic frequencies of *vgsc* L995F and N1570Y than *An. gambiae* s.s., *An. coluzzii* and *An. coluzzii-An. gambiae* hybrids (*P* < 0.05). Our study identified the L995F (kdr-west) allele in *An. melas* for the first time on the archipelago. *Anopheles melas* is described as an opportunistic vector which is highly anthropophilic and zoophilic, whereas *An. gambiae* s.s. has been described as a mostly anthropophilic species with a preference for human hosts [34]. This suggests that *An. melas* may be more likely to feed on animal hosts and encounter fewer ITNs than *An. gambiae* s.s*.* In addition, *Anopheles melas* also have higher tolerance for saline environments and are often found in salt marshes or mangroves [34]. This may also mean that *An. melas* encounter ITNs less frequently. In this case, *An. melas* would be under weaker selection pressure for *vgsc* resistance-conferring mutations than the other species. Furthermore, the extreme rates of *An. gambiae-An. coluzzii* hybridisation found on the Bijagós (> 20%) may facilitate increased propagation of *vgsc* mutations between *An. gambiae* s.s. and *An. coluzzii*, enabling these mutations to reach higher frequencies within these populations. However, a relatively small number of mosquitoes were sampled overall in this study, including only *n* = 11 *An. coluzzii* samples, making it difficult to draw definitive conclusions from these inter-species comparisons.

Overall, this study demonstrates the usability of high-throughput multiplex amplicon sequencing in screening insecticide resistance mutations in *Anopheles gambiae* s.l. mosquitoes. Four resistance-associated SNPs were identified in the Bijagós mosquito population. An additional eight non-synonymous SNPs were identified which have not previously been described. Phenotypic assays would be needed to determine whether these SNPs contribute to resistance. Importantly, insecticide resistance is only one component of effective vector control. Coverage, uptake and durability of ITNs, along with longevity of the insecticide, are all important factors which need to be measured [35].

 Limitations of this study include the relatively small sample of mosquitoes used in the analyses and that only loci that have been previously associated with insecticide resistance were surveyed. However, this is an easily scalable method, and additional genes can be added to the multiplex assay as these are identified. Multiplex amplicon sequencing allows the rapid screening of many loci, resulting in high-quality genomic data which can complement phenotypic assays of insecticide resistance and inform vector control policy.

## Conclusions

This study demonstrates the utility of multiplex amplicon sequencing for rapidly assessing the prevalence of insecticide resistance markers in mosquito populations. Furthermore, we provide the first assessment of molecular markers of insecticide resistance from multiple islands across the Bijagós Archipelago. Overall prevalence of these markers was low, but the presence of *vgsc* mutations L995F and N1570Y warrants further attention, particularly as vector control on the islands is limited to pyrethroid ITNs. The identification of additional molecular markers of insecticide resistance is required. This may be achieved through the combination of phenotypic bioassay data with genomic data, generated using either whole-genome sequencing or using high-throughput methods such as the multiplex amplicon sequencing technique used in this study.

### Supplementary Information

**Additional file 1: Table S1.** Target SNPs investigated which have been associated with insecticide resistance in the Culicidae family [18]. Genome and amino acid positions are labelled according to the position in *Anopheles gambiae* P4 genome. **Table S2.** Primers for insecticide resistance amplicon sequencing for the *Anopheles gambiae *sensu latu complex, adapted from Campos et al. [18]. **Table S3.** Barcodes BC1–BC10 were concatenated to the 5’ end of forward primers; BC11–BC20 were concatenated to the 5’ end of reverse primers.

## Data Availability

The raw sequence data generated and analysed during the current study are available in the European Nucleotide Archive (Project ID: PRJEB62886, Accession Numbers: ERS15575388–ERS15575673).

## Abbreviations

*ace1*: Acetylcholinesterase 1
CDC: Centers for Disease Control and Prevention
DDT: Dichlorodiphenyltrichloroethane
*gste2*: Glutathione S-Transferase Epsilon 2
IGS: Intergenic Spacer
INDELs: Insertions/Deletions
ITNs: Insecticide-Treated Nets
kdr: Knock-Down Resistance
RFLP: Restriction Fragment Length Polymorphism
*rdl*: Gamma-Aminobutyric Acid Receptor
SNP: Single Nucleotide Polymorphism
*vgsc*: Voltage-Gated Sodium Channel
WHO: World Health Organization
