# Supplementary material for: Genomic surveillance of Anopheles mosquitoes on the Bijagós Archipelago using custom targeted amplicon sequencing identifies mutations associated with insecticide resistance
Source: Parasit Vectors. 2024 Jan 4;17:10. doi: 10.1186/s13071-023-06085-5 (PMC10768400; doi:10.1186/s13071-023-06085-5)
Supplement: Supplementary file 1 — Additional file 1: Table S1. Target SNPs investigated which have been associated with insecticide resistance in the Culicidae family [18]. Genome and amino acid positions are labelled according to the position in Anopheles gambiae P4 genome. Table S2. Primers for insecticide resistance amplicon sequencing for the Anopheles gambiae sensu latu complex, adapted from Campos et al. [18]. Table S3. Barcodes BC1–BC10 were concatenated to the 5’ end of forward primers; BC11–BC20 were concatenated to the 5’ end of reverse primers. [file 13071_2023_6085_MOESM1_ESM.docx]

Additional Information

**Table S1:** Target SNPs investigated which have been associated with insecticide resistance in the *Culicidae* family [18]. Genome and amino acid positions are labelled according to the position in *Anopheles gambiae* P4 genome.

| **Gene** | **Chromosome** | **Position** | **SNP** | **Amino Acid change** |
| --- | --- | --- | --- | --- |
| *vgsc*-DIS6 | AgamP4_2L | 2391228 | G>C, T | V402L |
| *vgsc*-DIIS6 | AgamP4_2L | 2422575 | T >C | S970P |
| *vgsc*-DIIS6 | AgamP4_2L | 2422643 | A>G | I992M |
| *vgsc*-DIIS6 | AgamP4_2L | 2422651 | T >C | L995S |
| *vgsc*-DIIS6 | AgamP4_2L | 2422652 | A>T | L995F |
| *vgsc*-DIIS6 | AgamP4_2L | 2422657 | T>G | V997G |
| *vgsc*-DIIIS6 | AgamP4_2L | 2429617 | T>C | I1527T |
| *vgsc*-DIIIS6 | AgamP4_2L | 2429623 | T>G | F1529C |
| *vgsc*-DIIIS6 | AgamP4_2L | 2429745 | A>T | N1570Y |
| *vgsc-*DIVS5 | AgamP4_2L | 2430424 | G>T | A1746S |
| *vgsc-*DIVS5 | AgamP4_2L | 2430460 | G>T | D1758Y |
| *gste2* | AgamP4_3R | 28598166 | T>C | I114T |
| *gste2* | AgamP4_3R | 28598062 | C>G | L119V |
| *gste2* | AgamP4_3R | 28598057 | C>G,A | F120 L |
| *rdl* | AgamP4_2L | 25429236 | C>G | A296G |
| *rdl* | AgamP4_2L | 25429235 | G>T | A296S |
| *ace1* | AgamP4_2R | 3492074 | G>A | G280S |

**Table S2:** Primers for insecticide resistance amplicon sequencing for the *Anopheles gambiae sensu latu complex*, adapted from Campos *et al* (2022) [18]

| **Target Gene** | **Amplicon** | **Forward primer** | **Reverse Primer** | **Chromosome** | **Position*** | **Product Size (bp)*** |
| --- | --- | --- | --- | --- | --- | --- |
| *vgsc* | *vgsc-I* | ATTCGTTATTCTTCAGATGAACT | ATTCTCACCCGAAGTGC | AgamP4_2L | 2390813-2391328 | 517 |
|  | *vgsc-II* | GTTTTGCTAGCCTAATTGC | TGTCGGTTGAACGGATGCTATT | AgamP4_2L | 2422417-2422919 | 503 |
|  | *vgsc-III* | TTCATGGGAAAATTCACCAA | AATTAGTGCTCCAAACACAAAC | AgamP4_2L | 2429356-2429845 | 490 |
|  | *vgsc-IV* | CGAGCCATGGAATTTGT | TGATGTGATCCAGTTACAGA | AgamP4_2L | 2430093-2430593 | 501 |
| *ace1* | *ace1* | CTGGTGGTCAACACGGA | GAACAGTCCCGCATTGC | AgamP4_2R | 3491732-3492229 | 498 |
| *gste2* | *gste2* | GCCCGGATGAGATTCAT | GGCTAGCACAAACTTGC | AgamP4_3R | 28597778 -28598182- | 404 |
| *rdl* | *rdl* | CATTGCAATCATCACCATCA | CCAGCAGACTGGCAAATACC | AgamP4_2L | 25428861-25429373 | 500 |

*Position and product size in *An. gambiae P4.*

**Table S3**: Barcodes, BC1 to BC10 were concatenated to the 5’ end of forward primers. BC11 to BC20 were concatenated to the 5’ end of reverse primers.

| **Name** | **Barcode** | **Name** | **Barcode** |
| --- | --- | --- | --- |
| BC1N | CTATCACG | BC11N | ATGGCTAG |
| BC2N | TCCAGTGT | BC12N | GACTTGGT |
| BC3N | GATCAGTA | BC13N | TCGATCAC |
| BC4N | AGTGTCGG | BC14N | ACACGTCA |
| BC5N | GTAGCGCT | BC15N | CAATGTGC |
| BC6N | CATCTAAC | BC16N | GGGACTAC |
| BC7N | TACAGATC | BC17N | ACGTACTG |
| BC8N | CGTCTTGT | BC18N | TGATTGCC |
| BC9N | TATGATCA | BC19N | AACTCTAC |
| BC10N | GGTAGCTT | BC20N | TGACTCAA |
